# The Effect of Sleep Deprivation and Subsequent Recovery Period on the Synaptic Proteome of Rat Cerebral Cortex

**DOI:** 10.1007/s12035-021-02699-x

**Published:** 2022-01-05

**Authors:** Péter Gulyássy, Katalin Todorov-Völgyi, Vilmos Tóth, Balázs A. Györffy, Gina Puska, Attila Simor, Gábor Juhász, László Drahos, Katalin Adrienna Kékesi

**Affiliations:** 1 MTA-TTK NAP B MS Neuroproteomics Research Group, Research Center for Natural Sciences, Budapest, H-1117 Hungary; 2 Laboratory of Proteomics, Institute of Biology, ELTE Eötvös Loránd University, Budapest, H-1117 Hungary; 3 MTA-ELTE NAP Laboratory of Molecular and Systems Neurobiology, Institute of Biology, Hungarian Academy of Sciences and ELTE Eötvös Loránd University, Budapest, H-1117 Hungary; 4 ELTE-NAP Neuroimmunology Research Group, Institute of Biology, ELTE Eötvös Loránd University, Budapest, H-1117 Hungary; 5 Department of Anatomy, Cell and Developmental Biology, Institute of Biology, ELTE Eötvös Loránd University, Budapest, H-1117 Hungary; 6 Department of Ecology, University of Veterinary Medicine Budapest, Budapest, H-1078 Hungary; 7 MS Proteomics Research Group, Research Center for Natural Sciences, Budapest, H-1117 Hungary; 8 Department of Physiology and Neurobiology, ELTE Eötvös Loránd University, Budapest, H-1117 Hungary

**Keywords:** Sleep, Sleep deprivation, Recovery period, Synapse, Synaptosome, Proteomics

## Abstract

Sleep deprivation (SD) is commonplace in the modern way of life and has a substantial social, medical, and human cost. Sleep deprivation induces cognitive impairment such as loss of executive attention, working memory decline, poor emotion regulation, increased reaction times, and higher cognitive functions are particularly vulnerable to sleep loss. Furthermore, SD is associated with obesity, diabetes, cardiovascular diseases, cancer, and a vast majority of psychiatric and neurodegenerative disorders are accompanied by sleep disturbances. Despite the widespread scientific interest in the effect of sleep loss on synaptic function, there is a lack of investigation focusing on synaptic transmission on the proteome level. In the present study, we report the effects of SD and recovery period (RP) on the cortical synaptic proteome in rats. Synaptosomes were isolated after 8 h of SD performed by gentle handling and after 16 h of RP. The purity of synaptosome fraction was validated with western blot and electron microscopy, and the protein abundance alterations were analyzed by mass spectrometry. We observed that SD and RP have a wide impact on neurotransmitter-related proteins at both the presynaptic and postsynaptic membranes. The abundance of synaptic proteins has changed to a greater extent in consequence of SD than during RP: we identified 78 proteins with altered abundance after SD and 39 proteins after the course of RP. Levels of most of the altered proteins were upregulated during SD, while RP showed the opposite tendency, and three proteins (Gabbr1, Anks1b, and Decr1) showed abundance changes with opposite direction after SD and RP. The functional cluster analysis revealed that a majority of the altered proteins is related to signal transduction and regulation, synaptic transmission and synaptic assembly, protein and ion transport, and lipid and fatty acid metabolism, while the interaction network analysis revealed several connections between the significantly altered proteins and the molecular processes of synaptic plasticity or sleep. Our proteomic data implies suppression of SNARE-mediated synaptic vesicle exocytosis and impaired endocytic processes after sleep deprivation. Both SD and RP altered GABA neurotransmission and affected protein synthesis, several regulatory processes and signaling pathways, energy homeostatic processes, and metabolic pathways.

**Supplementary Information:**

The online version contains supplementary material available at 10.1007/s12035-021-02699-x.

## Introduction

Sleep is a general phenomenon in animals having neural network and occurs in all of the species investigated; however, its core function remains controversial. Sleep contributes to maintain physiological brain functions such as memory formation and storage and normal cognitive processes as decision-making, language, and categorization and restores performance, while it also serves multiple supplementary functions, e.g., affecting the immune system during infectious diseases and reducing calorie use enabling the restoration of depleted energy stores, and it also exerts a glymphatic function aimed to remove toxic substances [1–5]. Sleep benefits both declarative and nondeclarative memories, and during sleep, memories may be transformed into a more flexible representations that can be adopted for a host of other cognitive functions, including creative thinking and problem-solving [6].

Despite the lack of consensus about the underlying mechanisms, there are several lines of evidence supporting that the main roles of sleep are to restore the waking-induced performance deterioration and to serve a connectivity regulation function effectively restoring brain plasticity and proper cognition [7, 8]. In the past decades, two major hypotheses evolved concerning the overall function of sleep focusing on synaptic functions. One of them, coined as the synaptic homeostasis hypothesis (SHY) proposed by Tononi and Cirelli, states that wakefulness is accompanied by synaptic potentiation in a large fraction of cortical circuits resulting in a net increase in synaptic strength and the role of sleep is to downscale the overall synaptic strength in a non-Hebbian way to an energetically sustainable baseline level [9–11]. This homeostatic process leads to a net decrease of synaptic strength enabling further potentiation during subsequent waking; therefore, the sleep-promoted plasticity is a result of changes in the strength of synaptic connections. In contrast, the synaptic embossing theory states that long-term memory consolidation during sleep requires active morphological changes producing long-lasting modifications on the strength of synaptic connections [12]. Depending on the conditions of memory acquisition, instead of a general synaptic downscaling, a portion of neuronal circuits undergo experience-dependent upscaling of synaptic weights during sleep. Fluctuations of calcium levels in activated synapses activate multiple calcium-dependent kinases with a role in memory formation resulting in a pretranscriptional amplification of synaptic changes during SWS, which will be transcriptionally stored during REM sleep through CREB-dependent gene expression, triggering plasticity-related protein synthesis [13]. In this view, sleep not only restores the capacity of learning but also actively enhances memory encoding via influencing synaptic plasticity bidirectionally, promoting synaptic up- and downscaling simultaneously in distinct neuronal networks [14].

Sleep deprivation leads to overwhelming sleep pressure, sleep attacks, and involuntary microsleeps and degrades several aspects of neurocognitive performance. SD impacts on mood and induces cognitive impairments such as loss of attention, impaired multitasking, increased reaction times, impaired memory, poor emotion regulation, and cognitive decline. Higher cognitive functions are particularly vulnerable to sleep loss [15]. Furthermore, sleep deprivation is associated with obesity, diabetes, cardiovascular diseases, elevated stress responses, inflammatory reactions, increased neuronal apoptosis, elevated oxyradical levels, and a majority of neurodegenerative and mental disorders which are accompanied by sleep disturbances [16]. Severe sleep loss can be even lethal [17]. The main drivers of cellular damage and lethality are the reactive oxygen species (ROS) as prolonged sleep restriction leads to the accumulation of ROS and consequent oxidative stress, especially in the gut [18–20].

Proteomic approaches have contributed notably to the understanding of sleep-related molecular processes [21, 22]. Quantitative experiments performed on synaptic structures (e.g., isolated synaptosomes or postsynaptic densities) monitoring abundance alterations of proteins or posttranslational modifications have proven to be particularly attractive and have been applied to investigate circadian clock-driven mechanisms and physiological sleep [23–26]. Although the pathophysiological mechanisms of SD have been extensively studied in various models, unbiased proteomic studies are rare, and the molecular changes in the sleep-deprived brain are not completely understood.

Irrespective of hypotheses, sleep and sleep deprivation are known to have a profound effect on synaptic functions which have significant consequences on how the brain functions. Cortical oscillations and neuronal firing patterns mirror sleep homeostasis, and cortex and subcortical circuitry are believed to play distinct roles in the global control of vigilance states. Cortical networks are thought to be able to sense and integrate signals of sleep need arising as a result of a local accumulation of products of metabolism, such as adenosine [27]. Because SD-induced neurological changes might spread along the cortical synaptic networks, the aim of the present study was to describe the effect of SD and RP on the synaptic proteome of the rat cerebral cortex focusing on synaptic transmission.

## Materials and Methods

### Animals

Adult male Wistar rats (4 months old, weighing 350–400 g, purchased from Toxi-Coop Ltd., Budapest, Hungary) were used in the experiments. Animals were housed under standard laboratory conditions in a 12-h light-dark cycle (light was on from 09:00 AM to 09:00 PM) in air-conditioned rooms at 22 ± 2°C. Food and water were supplied ad libitum. Animals were subjected to 2 weeks of acclimatization before the experiments and all rats were shortly gently handled each day to become accustomed to the experimenter. Handling and experimentation on animals were performed conforming to the Code of Ethics of the World Medical Association (Declaration of Helsinki), the Council Directive 86/609/EEC, the Hungarian Act of Animal Care and Experimentation (1998, XXVIII), and local regulations for the care and use of animals for research. All efforts were taken to minimize the animals’ pain and suffering and to reduce the number of animals used in the experiment.

### Experimental Paradigm

**Figure 1:**
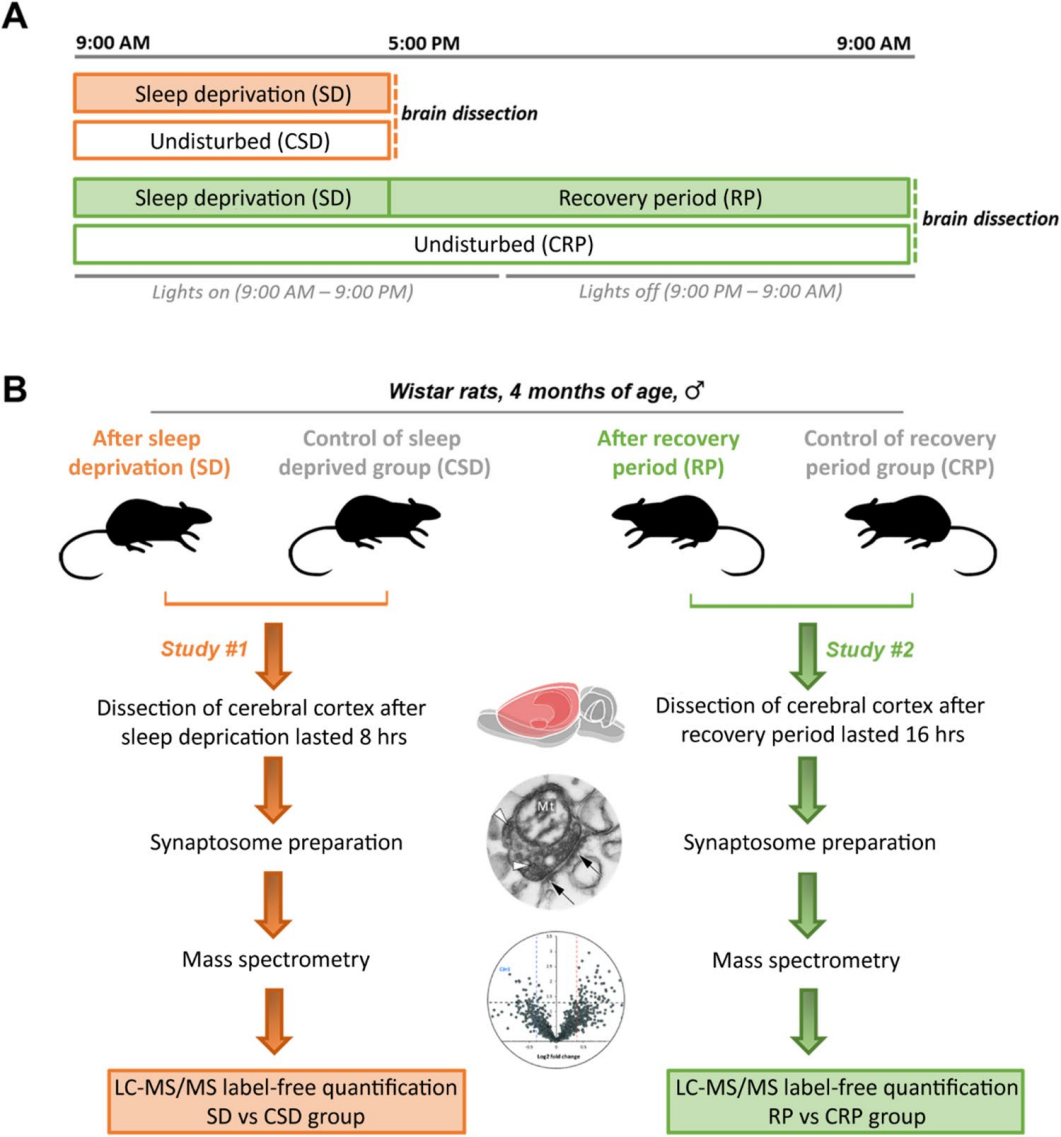
Experimental paradigm. Experimental paradigm for conducting sleep deprivation with subsequent recovery period and the proteomics workflow. **A** Timetable of the sleep deprivation procedure with subsequent recovery period and collection of brain samples. **B** Overview of the sample processing steps and the proteomics study design. In Study #1, proteomic comparison of synaptosomes was performed between sleep-deprived and the corresponding control group. Study #2 compared the synaptic proteome between the animals allowed for recovery period and the corresponding control group.

Sleep deprivation was carried out using the gentle handling method to minimize stress during the experiment. The deprivation started at 9:00 AM and lasted 8 h until 5:00 PM. The animals can be kept awake almost entirely during an 8-h deprivation without allowing microsleeps. In the sleep deprivation experiments, brains of the sleep-deprived (*n*=6) and undisturbed control (*n*=6) animals were removed right after the end of sleep deprivation. In the recovery period experiment, brains of the sleep-deprived (*n*=6) and control (*n*=6) animals were removed 16 h after finishing the sleep deprivation, on the next day at 9:00 AM (Figure 1). The 16-h recovery period extends across the entire dark phase, in which rats normally awake and the sleep pressure is expected to completely dissipate. Although local translation at synapses occurs very fast and influences the synaptic proteome expansively, translational processes might be necessary to restore the normal synaptic structure and function impaired by sleep deprivation. As synaptic proteins synthetized in the neuronal soma are actively transported along the axonal microtubules, several hours are needed until the newly synthetized proteins become integrated to the synaptic site. On the grounds of the slow re-supplement of synaptic proteins, in our experimental paradigm, 16 h of recovery period was allowed.

### Synaptosome Preparation

Synaptosome enrichment was performed following the protocols of Phillips et al. and Hahn et al. with minor modifications [28–30]. Briefly, rats were deeply anesthetized with i.p. urethane administration; the brains were quickly removed and washed in dry ice-cooled artificial cerebrospinal fluid (ACSF). Cerebral cortices from both hemispheres were dissected on a dry ice-cooled plate. Cortical tissues from the right hemispheres were homogenized in 1 ml of homogenization buffer containing 320 mM sucrose, 0.1 mM CaCl_2_, and 1 mM MgCl_2_, supplemented with Protease and Phosphatase Inhibitor Cocktails (Sigma-Aldrich, St. Louis, MO, USA) with 40 strokes in a Dounce-type glass homogenizer (Kontes Glass Co., Vineland, NJ, USA) using the small clearance pestle. The homogenates were adjusted to 1.25 M sucrose and 0.1 mM CaCl_2_ to a total volume of 5 ml. Five milliliters of 1 M sucrose solution was overlaid on them in a centrifuge tube and was centrifuged at 100,000 × *g* for 3 h in an SW-40 rotor (Beckman Coulter, Brea, CA, USA). The band at the interface was collected as the synaptosome fraction. The samples were diluted with 5 × volume of 0.1 mM CaCl_2_ and centrifuged at 15,000 × *g* for 20 min, and the pellets were precipitated with ice-cold acetone overnight. The next day, the samples were spun down, the acetone was removed, and the pellets were allowed to dry.

### Preparation of Whole Cerebral Cortex Homogenate

The brain of urethane-anesthetized rat was removed and washed with ice-cold ACSF, and the cortex was dissected on a dry ice-cooled plate. The tissue was mechanically homogenized in lysis buffer (7 M urea, 2 M thiourea, 4% (wt/vol) CHAPS, 20 mM Tris, 5 mM MgCl_2_, 50 mM dithiothreitol (DTT) using an IKA-Turrax blender (IKA-Werke, Staufen, Germany)) for 10 × 10 s at 15,000 rpm on ice. The homogenate was later sonicated (Ultrasonic Processor, Cole Palmer, Niles, IL, USA) for 10 × 10 s on ice.

### Western Blot Validation of Synaptosome Enrichment

The acetone-precipitated proteins from synaptosome samples and whole cortex homogenate were resuspended in lysis buffer (7 M urea, 2 M thiourea, 4% (wt/vol) CHAPS, 20 mM Tris, 5 mM MgCl_2_, 50 mM DTT) and sonicated on ice until completely dissolved. The protein concentration was determined using the 2D-Quant Kit (GE Healthcare, Little Chalfont, UK) according to the manufacturer’s instructions. Samples containing 20 μg protein amount were mixed with two-fold concentrated sample buffer (8% (wt/vol) SDS, 3% (wt/vol) DTT, 24% (vol/vol) glycerol, 0.2% (wt/vol) bromophenol blue, 100 mM Tris-HCl pH 6.8) to a total volume of 20 μl, and the samples were boiled at 96°C for 5 min. Proteins were separated on a discontinuous 10% polyacrylamide gel by Tricine-SDS gel electrophoresis and transferred onto a Hybond-LFP PVDF membrane (GE Healthcare). The blots were blocked with 5% (wt/vol) bovine serum albumin in Tris-buffered saline and 0.05% (vol/vol) Tween-20 (TBS-T) for 1 h. Subsequently, the membranes were incubated overnight in the blocking buffer with anti-Psd95 (1:1,500 dilution, Cat. no.: MA1-046, Thermo Fisher Scientific, Waltham, MA, USA) and anti-Syp (1:100 dilution, Cat. no.: sc-55507, Santa Cruz Biotechnology, Dallas, TX, USA) primary antibodies. The next day, the membranes were washed 4 × 5 min in TBS-T and were incubated with ECL Plex CyDye-conjugated anti-mouse IgG-Cy3 secondary antibody (1:2,500 dilution, GE Healthcare). After the washing steps in TBS-T, and then in TBS, the protein bands were visualized using a Typhoon TRIO+ (GE Healthcare) fluorescent laser scanner. The densitometric analyses were performed with the ImageJ program (http://imagej.nih.gov/ij/).

### Electron Microscopic Validation of Synaptosome Enrichment

The synaptosome preparations were fixed in 2% (wt/vol) formaldehyde (freshly depolymerized from paraformaldehyde) and 1% (wt/vol) glutaraldehyde in 0.1 M sodium cacodylate buffer (pH 7.4) for 1 h at room temperature. After washing thoroughly in 0.05 M Tris buffer, the samples were postfixed with a mixture of 0.5% (wt/vol) osmium tetroxide and 0.75% (wt/vol) potassium hexacyanoferrate for 1 h. Then, samples were stained with 2% (wt/vol) aqueous uranyl acetate for 30 min. Subsequently, the preparations were dehydrated through a graded series of ethanol and embedded in LR white resin (Sigma-Aldrich) according to the manufacturer’s instructions. Ultrathin sections (70 nm) were collected onto 300 mesh copper grids and were examined with a JEM-1011 electron microscope (JEOL, Tokyo, Japan) operating at 60 kV. Images were taken with an 11 megapixel Olympus Morada camera.

### Enzymatic Digestion and Sample Preparation for Mass Spectrometry

The precipitated samples were resuspended in lysis buffer (7 M urea, 2 M thiourea, 20 mM Tris, 5 mM Mg(Ac)_2_, 50 mM DTT) and were sonicated on ice until completely dissolved. The concentration was determined using the 2D Quant Kit (GE Healthcare), and the proteins were digested following the filter-aided sample preparation method published by Wisniewski et al. with minor modifications [31]. Briefly, samples with 150 μg of total protein were diluted with urea buffer (8 M urea, 100 mM Tris-HCl pH 8.5) to a total volume of 200 μl and were transferred to a Microcon YM-30 filter device (Merck Millipore, Billerica, MA, USA) and centrifuged at 14,000 × *g* for 15 min at room temperature. Then, 200 μl urea buffer was added to the samples and spun down again. For protein carbamidomethylation, 100 μl of iodoacetamide solution (50 mM iodoacetamide, 8 M urea, 100 mM Tris-HCl pH 8.5) was pipetted onto the filter and mixed at 450 rpm at room temperature for 3 min in a thermo-mixer. The samples were incubated for 45 min at room temperature in dark without mixing and at the end were centrifuged for 10 min. One hundred microliters of urea solution was added to the samples and spun down for 15 min, and this step was repeated twice. Subsequently, 100 μl of 50 mM NH_4_HCO_3_ was added, and the samples were centrifuged for 10 min, and this step was repeated twice. The proteins were recovered from the filter by a reverse spin of 1,500 × *g* for 3 min, and 100 μl of digestion solution (0.1% (wt/vol) RapiGest, 50 mM NH_4_HCO_3_) and trypsin (sequencing grade modified, Promega, Madison, WI, USA) in a 1:50 ratio was added. The samples were digested overnight at 37°C. The next day, the reaction was terminated by adding 4 μl formic acid (FA), and the samples were desalted on a Pierce C-18 spin column (Thermo Scientific, Sunnyvale, CA, USA) according to the manufacturer’s instructions and dried in a SpeedVac.

### LC-MS/MS Label-Free Quantification

Mass spectrometry-based label-free quantification of proteins present in the synaptosome samples was performed using a Maxis II ETD QqTOF (Bruker Daltonics, Bremen, Germany) coupled to an Ultimate 3000 nanoRSLC system (Dionex, Sunnyvale, CA, USA) controlled by Hystar v.3.2 (Bruker Daltonics). The samples were dissolved in 40 μl of 2% (vol/vol) acetonitrile (AcN) and 0.1% (vol/vol) FA, out of which, 6 μl was injected onto an Acclaim PepMap100 C-18 trap column (100 μm × 20 mm, Thermo Scientific). Sample desalting and pre-concentration were performed with 0.1 % (vol/vol) trifluoroacetic acid for 8 min with a flow rate of 5 μl/min. Peptides were separated on an ACQUITY UPLC M-Class Peptide BEH C18 column (130 Å, 1.7 μm, 75 μm × 250 mm, Waters, Milford, MA, USA) at 48°C using a flow rate of 300 nl/min. Eluent A was 0.1% (vol/vol) FA, while eluent B was AcN, 0.1% (vol/vol) FA. The gradient started with 4% B from 0 to 11 min, followed by a 120-min gradient to 50% B, and then the concentration of the solvent B was elevated to 90% in 1 min and kept there for 10 min. To avoid carryover, a blank was run after each sample. Sample ionization was achieved in positive electrospray ionization mode via a CaptiveSpray nanoBooster ion source. The nanoBooster pressure was 0.2 bar, the capillary voltage was set to 1,300 V, the drying gas was heated to 150°C, and the flow rate of the drying gas was 3 l/min. External mass calibration was performed via direct infusion using a low concentration tuning mix (Agilent Technologies, Santa Clara, CA, USA), and internal mass calibration was performed via lock mass for each run using sodium formate. The ion transfer parameters were set as follows: prepulse storage 10 μs, collision transfer 10 μs, quadrupole ion energy 5 eV, Funnel 1 RF 400 Vpp, and Multipole RF 400 Vpp. The collision RF was set to 1,200 Vpp, and the ion transfer time was 120 μs. The MS spectra were recorded with a fixed cycle time of 2.5 s over the mass range of m/z 150–2,200 at 3 Hz with a minimal precursor mass of 322 m/z. The CID was performed at 16 Hz for abundant precursors and at 4 Hz for ones of low abundance. Singly charged peptides were excluded from the analysis, and only multiply charged peptides were chosen for fragmentation. The collision energy for precursor signals was set automatically based on the isolation m/z, isolation mass range width, and charge state of the ion, according to the manufacturer’s recommendations. An active exclusion of 2 min after 1 spectrum was used except if the intensity of the precursor was elevated threefold. For protein content analysis, raw data were recalibrated using the Compass DataAnalysis software 4.3 (Bruker Daltonics). The samples were matched with the *Rattus norvegicus* SwissProt database using the Mascot server v.2.5 (Matrix Science, London, UK). The parameters for the Mascot search were set as follows: trypsin as the enzyme, max. 2 missed cleavages were allowed, and cysteine carbamidomethylation as fixed and methionine oxidation as variable modifications. Precursor tolerance and MS/MS tolerance were set to 7 ppm and 0.05 Da, respectively. Decoy database was generated by Mascot, and the false discovery rate was less than 1% in every search result. Proteins with a minimum of two identified unique peptides were accepted. Label-free quantification was performed using MaxQuant software version 1.5.3.30. LC-MS/MS runs were aligned using the “match between runs” feature (match time window 0.8 min, alignment time window 15 min). The following requirements were set: minimum peptide ratio count 2 and “unique + razor” peptide for quantification. In quantitative analysis, only the peptides without modifications were taken into consideration.

### Functional Classification

Significantly altered proteins were categorized manually according to the UniProt (http://www.uniprot.org) and Gene Ontology (http://geneontology.org) databases. For proteins having multiple roles, only the most relevant functions were assigned. Bioinformatic pathway analysis was performed by Pathway Studio 11.0 Platform (Elsevier Life Science Solutions).

## Results

### Validation of the Synaptosome Purification Process

**Figure 2:**
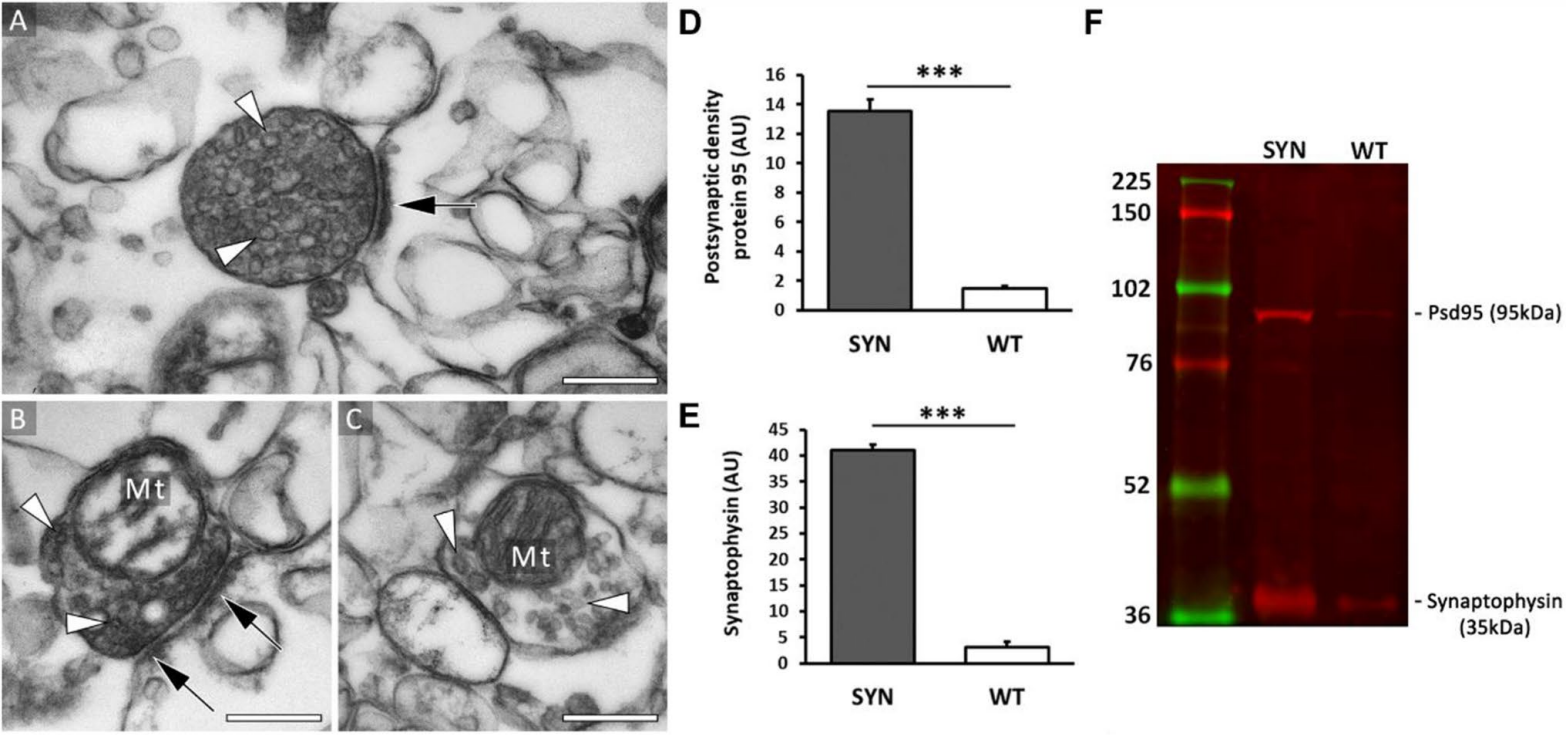
Validation of synaptosome sample. Representative electron micrographs showing characteristic synaptosomes (**A**, **B**, **C**). White arrowhead, synaptic vesicle; black arrow, postsynaptic density (PSD); Mt, mitochondrion. Scale bar: 0.5 μm in all panels. Western blot validation of the enrichment of the marker proteins postsynaptic density protein (Psd95) (**D**) and synaptophysin (**E**) in the synaptosome samples (SYN) compared to the whole, unfractionated cortical tissue homogenate (WT) (**F**). *n*=4, means ± SEM, two-sample Student’s *t*-test, ***P < 0.001. Normalization by total protein staining with CBB.

A small portion of the synaptosome samples was used to validate the enrichment of synaptic structures by electron microscopy. The samples consisted of sealed particles, and most of them showed characteristic features of a synaptosome. Many structures showed intact presynapse morphology with large pools of synaptic vesicles with the presence of the postsynaptic density attached to the presynaptic compartment as an electron-dense layer. A large portion of synaptosomes contained presynaptic mitochondria, but extrasynaptic mitochondria were not observed. A high number of empty, sealed particles were visible, which are thought to be synaptosomes that lost their internal content through the homogenization process before the membranes were resealed in the isoosmotic medium. Recognizable contaminating structures (e.g., nucleus, endoplasmic reticulum (ER)) were not visible in any of the images (Figure 2).

The enrichment of the synaptic protein pool in the synaptosome samples was validated with western blot by assessing the levels of two well-accepted synaptic marker proteins, synaptophysin and Psd95. The unfractionated cortical homogenate was compared to the cortical synaptosome fraction, and the immunoreactivity of the synaptic vesicle/presynaptic membrane-localized synaptophysin and the postsynaptically localized scaffold protein Psd95 were examined. The synaptic proteome contained 9.09 ± 1.00 times higher amount of synaptophysin and 13.35 ± 0.93 times higher amount of Psd95 as the same quantity of whole, unfractionated cortical proteome, proving the efficiency of the enrichment of synaptic structures (Figure 2).

### Quantitative Alterations in the Synaptic Proteome due to Sleep Deprivation and Subsequent Recovery Sleep

Altogether, 24 cortical synaptosome samples were analyzed from four groups (after sleep deprivation (SD, *n*=6), after recovery period (RP, *n*=6), corresponding control groups (CSD and CRP, *n*=6 each)). On average, 67,500 MS/MS spectra were acquired per sample, and the analyses of the 24 samples led to the detection of 1,475 proteins. During the quantification with MaxQuant, only those proteins were taken into account, which were quantifiable in at least five samples per group. From the sleep-deprived and corresponding control animals, 1,120 and 1,153 proteins were quantified at least in five samples, respectively, among which, 1,061 proteins were present in both experimental groups. In the recovery period group, 1,092 proteins were quantifiable at least in five samples, while 1,085 were in the corresponding controls, allowing us to compare the abundance of 1,009 common proteins between the two groups. The quantitative analysis of the protein abundances between the different groups revealed 78 significantly altered proteins in the SD and CSD comparison. Among them, 56 proteins showed significantly increased protein levels, while 22 proteins were decreased. The differences in the abundances were in the range of +3.86-fold and −1.82-fold. Comparison of protein abundances between the group sacrificed after the recovery period (RP) and its corresponding control group (CRP) uncovered 39 proteins with significantly altered levels, among which, 15 and 24 proteins were upregulated and downregulated, respectively. The differences in abundances were between the range of +1.82-fold and −1.99-fold.

**Figure 3:**
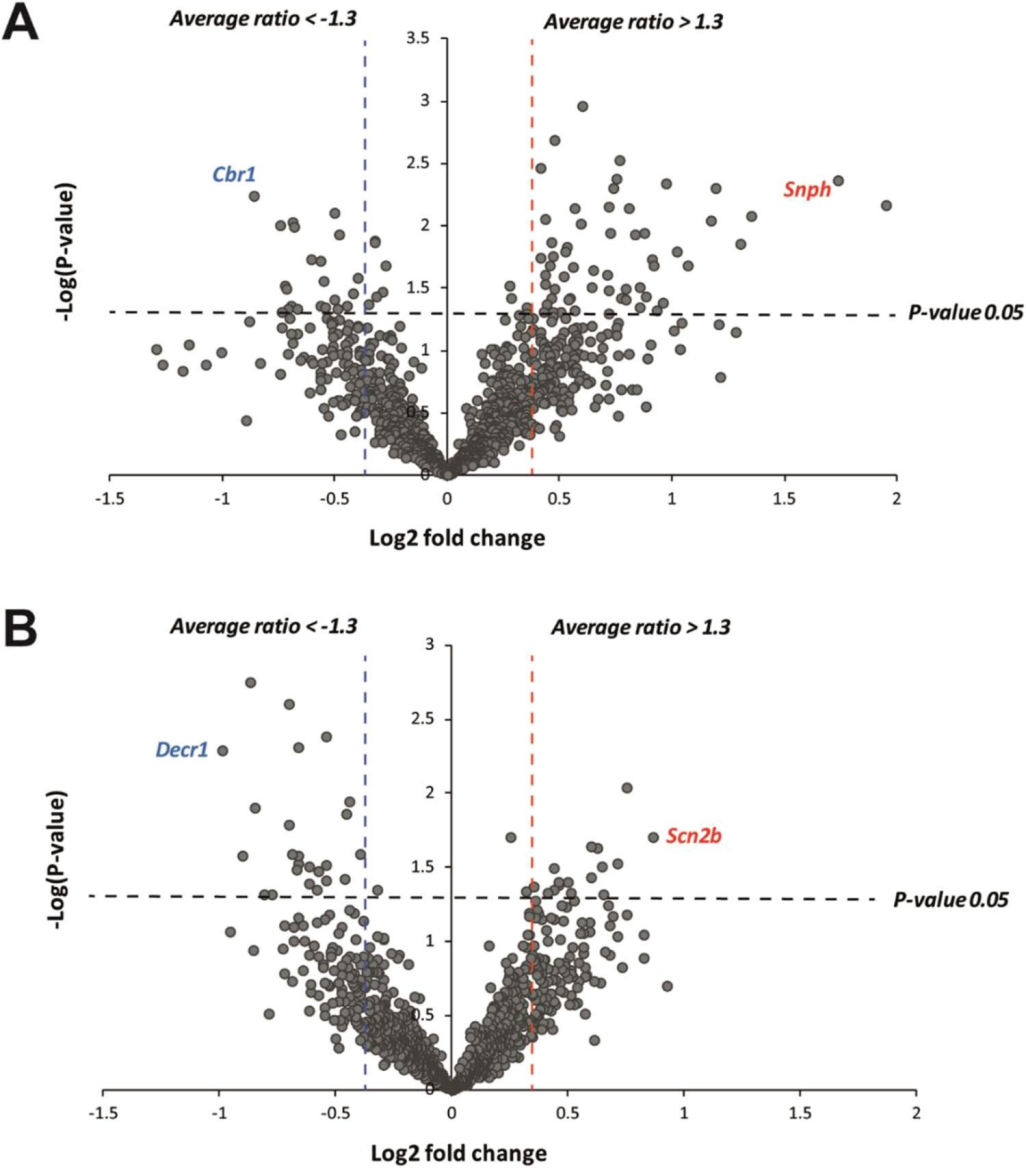
Magnitude and significance of protein abundance alterations. Volcano plots illustrate the magnitude and significance of the protein level differences between the sleep deprivation (**A**) and recovery period (**B**) groups in comparison with their respective controls. The −log10(*P*-value) is plotted against the log2(fold change: SD/CSD or RP/CRP). The non-axial vertical lines denote ±1.3-fold change, while the non-axial horizontal line denotes the *P* = 0.05 significance threshold (prior to logarithmic transformation). According to these two parameters, the most significantly altered proteins are highlighted with their gene names.

To graphically represent the significance and magnitude of protein changes, volcano plots were constructed as follows: log10(*P*-value) vs. either log2(fold change of SD/CSD) (Figure 3A) or log2(fold change of RP/CRP) (Figure 3B). Dots above the non-axial horizontal line represent proteins with significantly different abundances (*P* < 0.05). Dots to the left of the left non-axial vertical blue line indicate less than −1.3-protein fold changes of either SD or RP groups compared to their respective controls, while dots to the right of the right red non-axial vertical line label indicate greater than +1.3-protein fold changes in the same comparison.

### Functional Clustering of Significantly Altered Proteins

**Figure 4:**
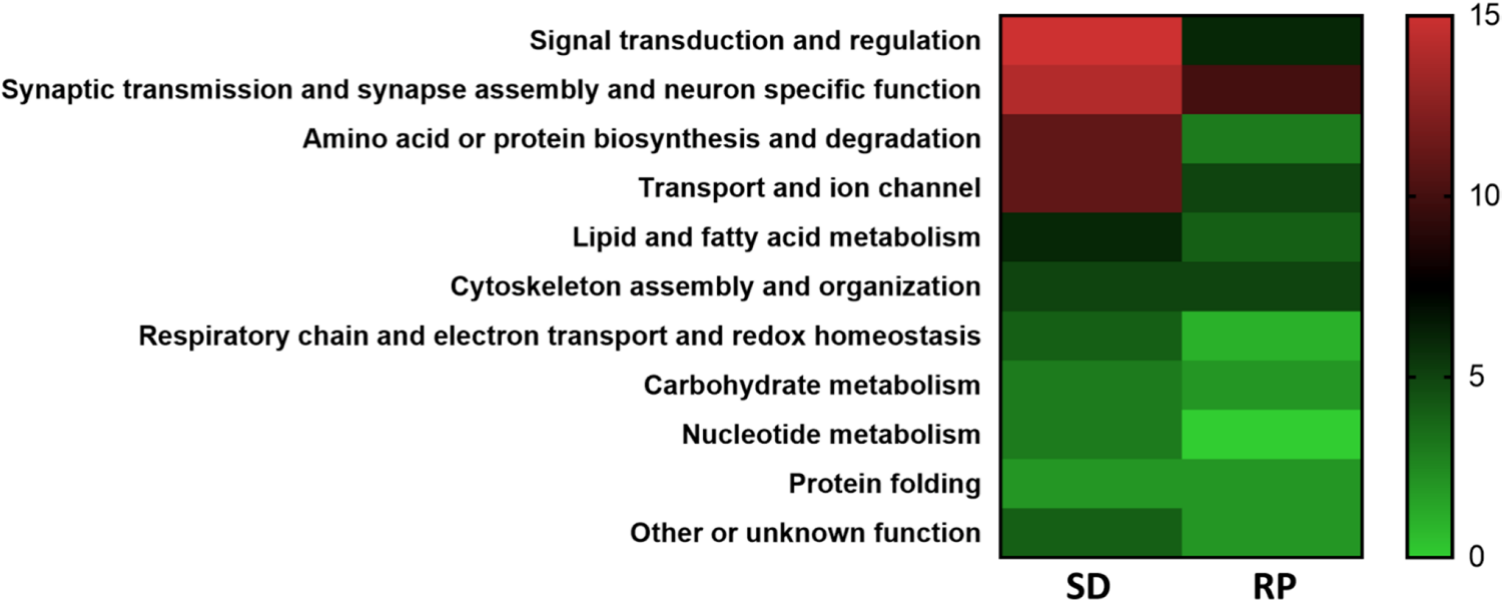
Functional clusters of altered proteins. Heat map of the functional clusters of the significantly altered proteins after sleep deprivation (SD) and recovery period (RP). (The color gradient represents the count of the significantly altered proteins).

Significantly altered proteins from the two sets of comparisons were clustered, based on their cellular functions to 11 functional groups as follows: signal transduction and regulation (*n*_SD/CSD_=15, *n*_RP/CRP_=6); lipid and fatty acid metabolism (*n*_SD/CSD_=6, *n*_RP/CRP_=4); amino acid or protein biosynthesis and degradation (*n*_SD/CSD_=11, *n*_RP/CRP_=3); respiratory chain, electron transport, and redox homeostasis (*n*_SD/CSD_=4, *n*_RP/CRP_=1); carbohydrate metabolism (*n*_SD/CSD_=3, *n*_RP/CRP_=2); nucleotide metabolism (*n*_SD/CSD_=3); transport and ion channel (*n*_SD/CSD_=11, *n*_RP/CRP_=5); synaptic transmission, synapse assembly, and neuron-specific function (*n*_SD/CSD_=14, *n*_RP/CRP_=10); cytoskeleton assembly and organization (*n*_SD/CSD_=5, *n*_*RP*/CRP_=5); protein folding (*n*_SD/CSD_=2, *n*_RP/CRP_=2); and other or unknown function (*n*_SD/CSD_=4, *n*_RP/CRP_=2) (Figure 4, Table 1). The localization, biological processes, *P*-value, and fold change parameters of the significantly changed proteins are presented in supplementary tables (Supplementary Table 1 for sleep deprivation and Supplementary Table 2 for recovery period).

**Table 1:**
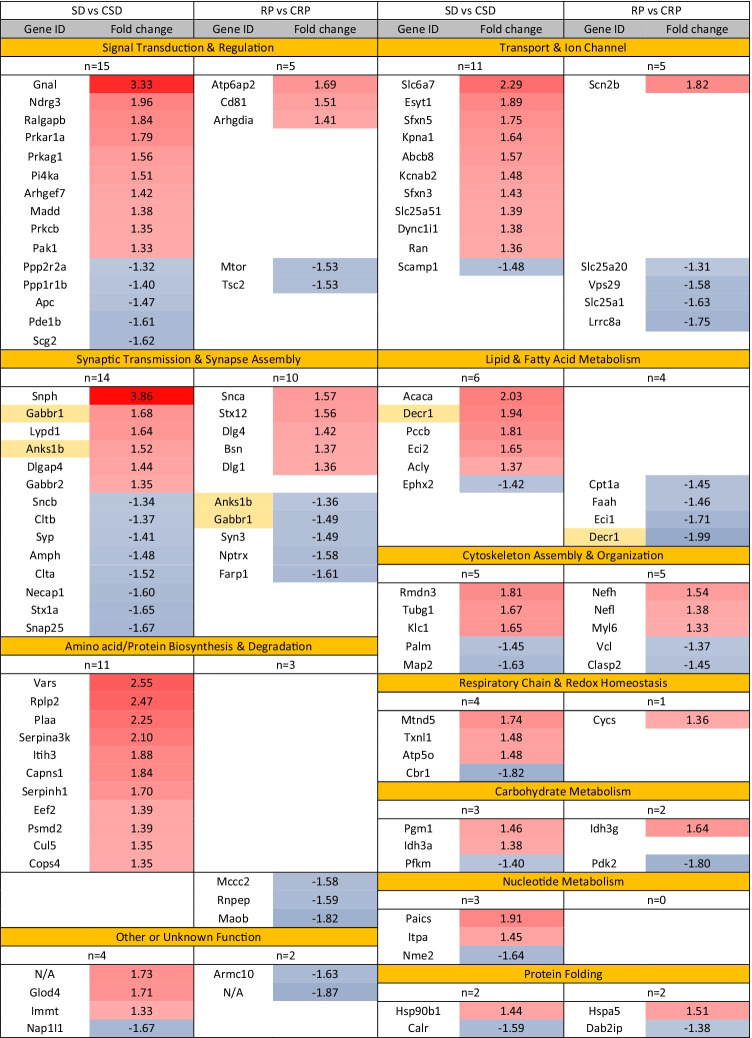
Functional clusters of altered proteins. Comparison of the significantly altered synaptic proteins in the cerebral cortex of rats after sleep deprivation and recovery period assigned to functional clusters. The red and blue color gradients are used to show the degree of increase or decrease, respectively, of the abundance of each protein compared to the corresponding control group. Gene names with yellow highlight indicate the common protein changes between SD and RP.

### Bioinformatics Analysis

**Figure 5:**
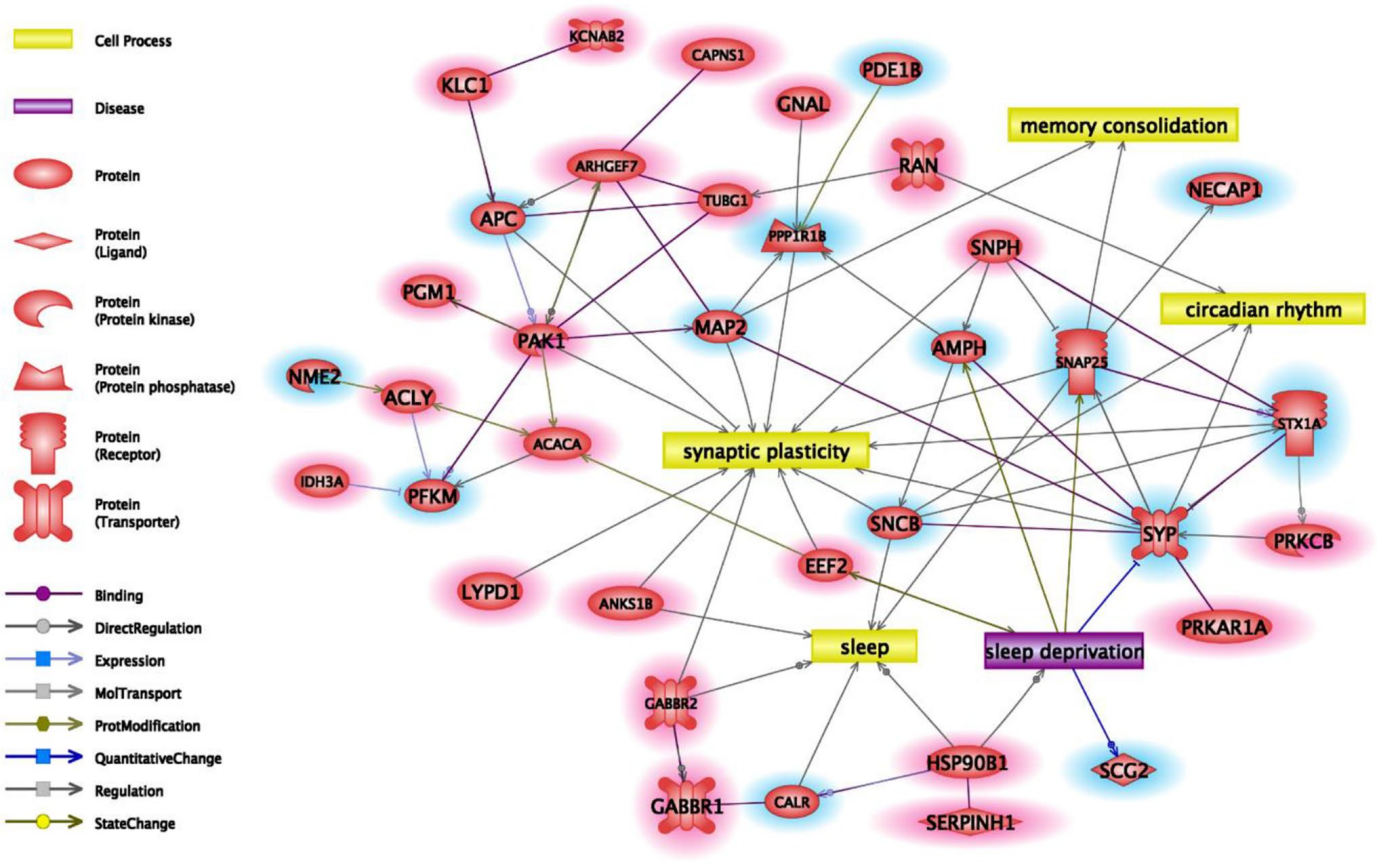
Connections of altered proteins after sleep deprivation. Connections of significantly altered synaptic proteins after sleep deprivation with each other and with sleep and sleep-related cellular processes. Red/blue highlights indicate the sleep deprivation-induced increase or decrease in abundance.

The direct interaction network of the significantly altered proteins after sleep deprivation and recovery period was constructed using the Elsevier Pathway Studio software and database. The interaction network of synaptic proteins significantly altered after sleep deprivation contains 22 proteins with increased and 14 proteins with decreased abundance. Among them, several proteins are related to the molecular processes of synaptic plasticity (*n*=13), sleep (*n*=6), circadian rhythm (*n*=3), and memory consolidation (*n*=2), while 6 proteins have a known relation with sleep deprivation (Figure 5).

**Figure 6:**
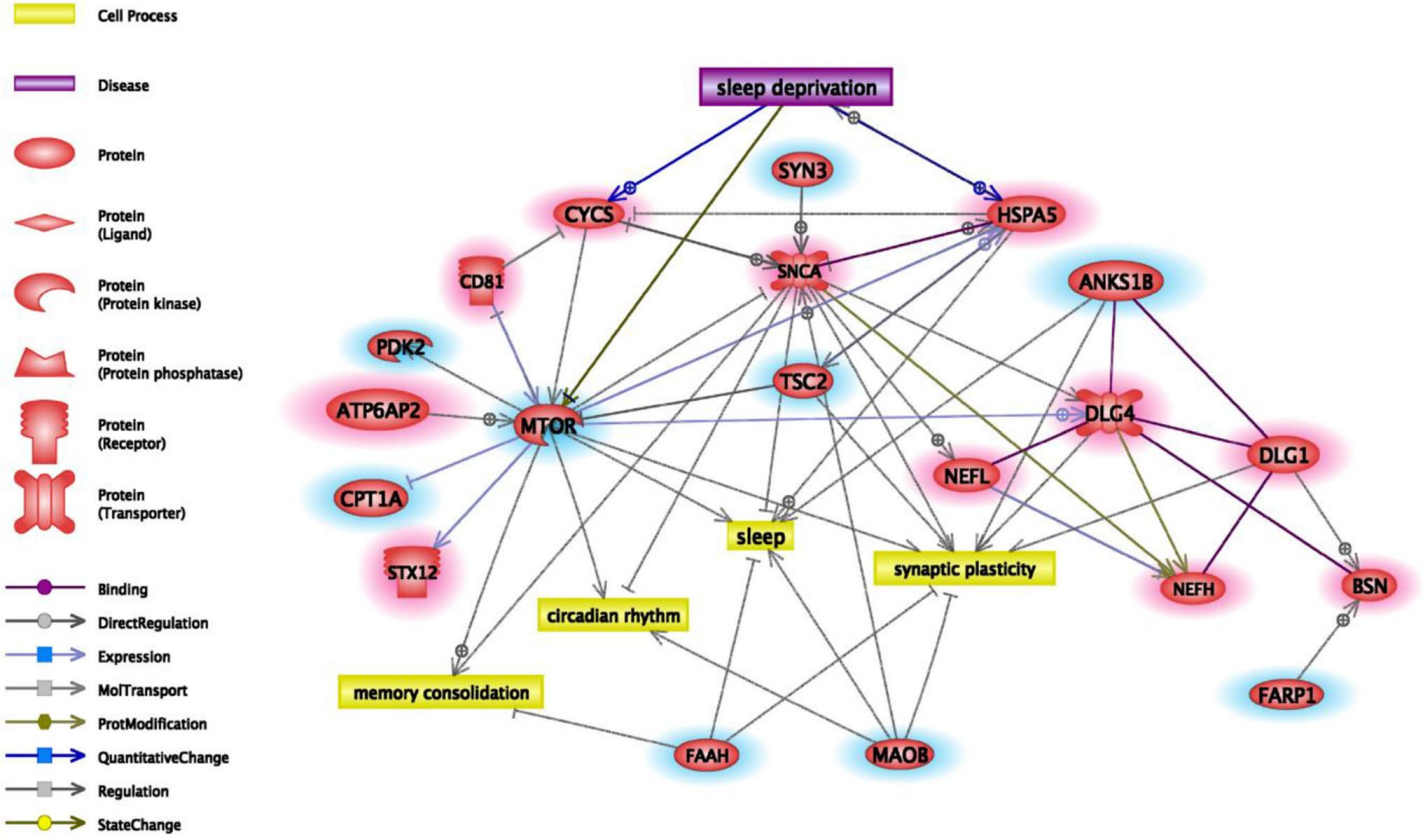
Connections of altered proteins after recovery sleep. Connections of significantly altered synaptic proteins after recovery period with each other and with sleep and sleep-related cellular processes. Red/blue highlights indicate the recovery sleep-induced increase or decrease in abundance.

The interaction network of significantly altered synaptic proteins after recovery period contains 11 proteins with increased and 9 proteins with decreased abundance. Among them, some proteins are related to the cellular processes of synaptic plasticity (*n*=6), sleep (*n*=4), circadian rhythm (*n*=3), and memory consolidation (*n*=3), while 3 proteins have a known relation with sleep deprivation (Figure 6).

## Discussion

Based on this study, we conclude that the synaptic protein expression pattern has changed to a greater extent in consequence of sleep deprivation (two times more protein showed significant changes in abundance) than during the recovery period. The levels of most of the altered proteins (70%) were upregulated during sleep deprivation, while protein changes after recovery period showed an opposite tendency (60% of the proteins were downregulated).

In the understanding of the underlying molecular mechanisms, we set a high value on the proteins which were detected to come through abundance alterations on account of both sleep deprivation and recovery period. Three proteins were identified with an abundance change in the opposite direction after sleep deprivation and recovery period. The metabotropic GABA_B_ receptor (Gabbr1), the ankyrin repeat and sterile alpha motif domain-containing protein 1B (Anks1b), and the 2,4-dienoyl-CoA reductase, mitochondrial enzyme (Decr1), showed elevated protein abundance after sleep deprivation; however, all three proteins were downregulated after the recovery period.

After sleep deprivation, syntaphilin (Snph) showed the highest protein level increase, while carbonyl reductase [NADPH] 1 (Cbr1) showed the highest decrease in protein abundance. After recovery period, sodium channel subunit beta 2 (Scn2b) showed the most prominent protein level increase and 2,4-dienoyl-CoA reductase, mitochondrial (Decr1), showed the highest decrease.

The functional cluster analysis showed that most of the altered proteins are related to signal transduction and regulation, synaptic transmission and synaptic assembly, protein and ion transport, and lipid and fatty acid metabolism, suggesting the importance of these biological processes in sleep.

In the discussion, we prioritize proteins having either specific synaptic functions or described roles in sleep-related processes.

### Sleep Deprivation and Recovery Period Have a Wide Impact on Synaptic Neurotransmission-Related Proteins

In our study performed on synaptosome samples, we were able to analyze the abundance alteration of several proteins having a direct role in synaptic transmission, among which the majority proved to be sleep-related. Note that our applied synaptosome isolation approach was not able to distinguish between different types of synapses and therefore the sample contained a pool of virtually all types of cortical synapses in a physiological ratio.

Moreover, synaptosome preparations always contain contamination to some extent from other subcellular organelles and non-neuronal cell types. The applied procedure for synaptosome isolation is capable to enrich both the presynaptic and postsynaptic components of the synapses and to deplete the sample from extrasynaptosomal mitochondria and oligodenrocyte-derived structures. However, it contains a relatively high astrocyte-derived contamination [30]. Since there is an overlap in the protein expression profile of astrocytes and neurons, in the case of proteins present in both cell types, a detected quantitative alteration cannot be attributed to a given cell type. In the discussion, we presume that all the detected quantitative protein alterations are consequences of changes in the synapses, but it cannot be ruled out that astrocytic alterations contribute to the shift in the proteome profile.

**Figure 7:**
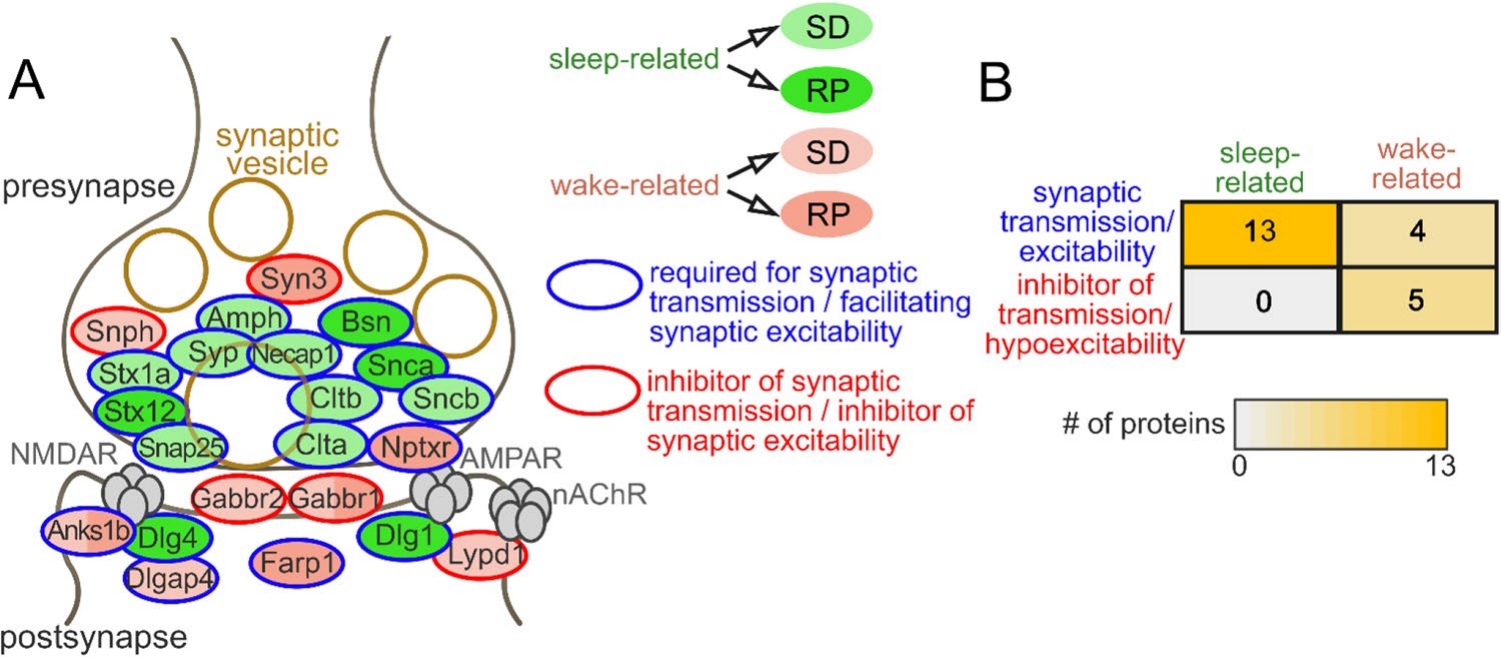
Overview of the transmission-related proteins. Overview of sleep-wake cycle-regulated proteins with primarily synaptic transmission-related functions. **A** Altered proteins were located to their known sub-synaptic compartment on this schematic, theoretical synapse incorporating proteins of distinct synapse sub-types (i.e., excitatory, inhibitory, and modulatory). According to their uncovered overall level changes and literature data on their specific functions, proteins were assigned to opposing groups (i.e., sleep/wake-related, facilitator/inhibitor of synaptic transmission and excitability). In this binary approach, a given protein has been labeled as sleep-related if its level was decreased after SD or increased after RP compared to their respective control conditions, while wake-related proteins were defined as ones with level changes in the opposite direction. **B** Summarization of this clustering pointing to the markedly distinct functional role of sleep and wake-related synaptic proteins.

We decided to focus specifically on proteins directly involved in synaptic transmission because the overall re-adjustment of cerebral cortical synaptic strength has been proposed to be a fundamental phenomenon during sleep (e.g., as postulated by the SHY theory), and hence, one might expect a profound re-organization of this component of the synaptic proteome. Generally, an enrichment was apparent in favor of synaptic transmission- and excitability-related proteins among the sleep-related ones (Figure 7). On the other hand, the wake-related proteins are associated with synaptic excitability and inhibition in a nearly equal ratio (Figure 7).

#### Neurotransmitter Receptors

The metabotropic GABA_B_ receptor is a member of the G protein-coupled receptors and located on presynaptic, postsynaptic, and extrasynaptic membranes [32]. The GABA_B_ receptors of the presynaptic neuron are mostly present at extrasynaptic sites and enriched on the rim of glutamate release sites, while the postsynaptic receptors are located on dendritic shafts and extrasynaptic membrane of spines [33]. In general, GABAergic signaling is important for normal sleep, and GABA advances sleep onset and prolongs total sleep; moreover, GABA_B_ receptors are involved in sleep-waking regulation [34]. Despite the GABA_B_ receptor agonist GHB (γ-hydroxybutyric acid) is an approved therapeutic drug for narcolepsy with cataplexy, the exact role of GABA_B_ receptors in sleep is poorly understood [35, 36]. Human studies with GHB showed improvements in sleep architecture deficits and subjective sleep [37]. Moreover, GABA_B_ receptor antagonists decrease overall sleep in rats, with particularly detrimental effects on slow-wave sleep [38]. When GABA_B_ receptor antagonists are infused in the thalamus of freely moving cats, slow-wave EEG activity and deep slow-wave sleep are decreased, while light slow-wave sleep is increased [39]. GABA_B_ receptor agonists have minimal effect on rapid eye movement sleep while increasing slow-wave sleep, and the circadian rhythm and slow-wave sleep-related gene expression [40, 41]. Genetically modified mice devoid of functional GABA_B_ receptors show a greatly altered sleep-wake distribution and sleep architecture. The overall lacking of GABA_B_ receptors leads to less fragmented sleep and a reduced EEG activity in the theta frequency range [42]. However, the homeostatic regulation of sleep after 6-h-long sleep deprivation is not affected in mice lacking GABA_B_ receptor subunits. In contrast, in Wistar rats, a 12-h-long total sleep deprivation increases the expression of GABA_B_ receptors and their heterodimerization with mGlu1αR in the CA1 region of the hippocampus demonstrated with a combination of western blotting, peroxidase immunocytochemistry and co-immunoprecipitation [43]. Consistent with the latter results, we detected with label-free mass spectrometry that both Gabbr1 and Gabbr2 subunits were upregulated (+1.68 and +1.35-fold change, respectively) after 8 h of sleep deprivation in the cortex. Additionally, we detected a reduced Gabbr1 level after recovery period (−1.49-fold change).

#### Scaffold and Synaptic Organizer Proteins

The ankyrin repeat and sterile alpha motif domain-containing protein 1B (Anks1b) is one of the most abundant proteins at neuronal synapses serving as a scaffolding protein with several protein-protein interaction domains and playing a broad role in cell signaling, gene expression, and synaptic plasticity regulation [44, 45]. *Anks1b* is a crucial gene for human development, in which loss-of-function variants lead to the reduced synaptic expression of the NMDAR subunit GluN2B, impaired NMDA-dependent LTP and LTD, and neurodevelopmental disorders [46, 47]. Anks1b isoforms are enriched in microsomes derived from light membrane fractions and at postsynaptic densities, where they bind to several types of proteins, including scaffolding proteins (Psd95 and Dlgap93), synaptic vesicle proteins (Snx27 and AP-2), enzymatic switches (SynGAP1 and AAK1), and other membrane proteins (NMDAR and contactin-1) [46]. The AIDA-1d isoform is a synapse-to-nucleus messenger by linking NMDAR activity to global changes in protein synthesis via altering nucleolar numbers. AIDA-1d couples to NMDAR via the scaffold protein Psd95, and stimulation of the NMDARs results in a Ca^2+^-independent translocation of AIDA-1d to the nucleus. Prolonged neuronal stimulation results in an AIDA-1d-dependent increase in nucleolar number and protein synthesis, which may be essential for neuronal survival, and may also help implement changes in protein synthesis during periods of persistent synaptic activity [48, 49]. In our proteomics analysis, we detected an increased Anks1b level (+1.52-fold change) after sleep deprivation, suggesting upregulated regulatory processes in synapses. In contrast, the abundance of Anks1b after recovery period was downregulated (−1.36-fold change).

The MAGUK family members Disks large homolog 1 and 4 (Dlg 1 and 4, alternative names: SAP97/synapse-associated protein 97 and Psd95/postsynaptic density protein 95) proteins were upregulated on account of recovery period (+1.36 and +1.42-fold change, respectively). Both proteins are multidomain scaffolding proteins providing a platform for the recruitment, trafficking, clustering and stabilization of receptors (NMDARs and AMPARs), ion channels, and signaling molecules to the postsynaptic density in excitatory synapses. Both proteins are essential contributors to the formation of cell polarity, glutamatergic transmission, synaptic plasticity, and dendritic spine morphogenesis. Dlg4 regulates synaptic strength and learning and has a pivotal role in the stabilization of synaptic structure after stimulation [50, 51]. Dlg1 isoforms impair LTP but enhance LTD via independent isoform-specific mechanisms. The α-isoform selectively regulates the synaptic pool of AMPA receptors, while the β-isoform regulates the extrasynaptic pools of both AMPA and NMDA receptors [52]. The upregulation of the two proteins under recovery period indicates extensive realignment in the structures of glutamatergic synapses. Another adaptor protein of the glutamatergic postsynaptic density, the Disks large-associated protein 4 (Dlgap4), also showed elevated abundance (+1.44-fold change), but in the consequence of sleep deprivation. Dlgap proteins not only induce the enrichment of Dlg4/PSD95 at the plasma membrane but also control its degradation indirectly. Upon dissociation of Dlgap from the Dlg4, both proteins are ubiquitinated and degraded [53]. The upregulation of Dlgap4 indicates that the molecular processes leading to the Dlg-mediated realignment of the excitatory synapse begin even under the sleep deprivation period.

The presynaptic protein bassoon is a large scaffold protein localized at the active zone in both excitatory and inhibitory synapses and takes part in a wide range of molecular processes. Bassoon participates in the formation of piccolo-bassoon transport vesicles that transport a range of proteins along axons to nascent synaptic terminals, involved in the tethering and priming of synaptic vesicles, regulating the presynaptic integrity and proteostasis, and mediates synapse-to-nucleus signaling [54]. Its upregulation during recovery period (+1.38-fold change) is expected to have a wide impact on presynaptic structure and function.

Non-scaffold proteins which might contribute to synaptic remodeling are the FERM, RhoGEF and pleckstrin domain-containing protein 1 (Farp1), and neuronal pentraxin receptor (Nptxr). Both proteins are downregulated at the end of recovery period (−1.61 and −1.58-fold change, respectively) and contribute to distinct but complementary mechanisms. While Farp1 increases synapse number and modulates synapse and dendrite spine development and morphology downstream of synaptic adhesion, Nptxr organizes both excitatory and inhibitory synapses without affecting synapse numbers by mechanisms depending on the activity of the neurotransmitter receptors [55, 56].

#### Neurotransmitter Release and Endocytosis

Although synaptosomes are known to contain increased neurotransmitter quanta after REM sleep deprivation, in our experiment, several proteins that contribute to neurotransmitter exocytosis showed decreased abundance after total sleep deprivation [57]. The coexistence of downregulation of key exocytosis-related proteins and the elevated neurotransmitter levels in the brain might arise from the shift of the exocytosis mode (e.g., kiss-and-run or ultrafast) or from the increase of neurotransmitter quanta per vesicle.

Synaptosomal-associated protein 25 (SNAP25) is a component of the SNARE complex, which has specific roles in various stages of neurotransmitter exocytosis from the synaptic vesicle priming and docking to the fusion pore formation. Point mutations in SNAP25 result in abnormal recycling of synaptic vesicles and disrupted synaptic connectivity and circadian rhythm impairment [58, 59]. SNAP25 shows decreased protein abundance in the wake-promoting area of the basal forebrain in rat after sleep deprivation [60]. In line with these data, in our experiments, sleep deprivation induced decreased (-1.67-fold change) synaptic protein level of SNAP25 in the cerebral cortex suggesting a downregulated synaptic transmission. Syntaxins are transmembrane Q-SNARE proteins among which syntaxin 1a (Stx1a) and 1b (Stx1b) are the major isoforms in the brain. Stx1a is a binding partner of the R-SNARE synaptobrevin and the Q-SNARE SNAP25 in the formation of the core SNARE complex [61]. Sleep deprivation leads to a decreased abundance of syntaxin 1b in parallel with SNAP25 (−1.65-fold change). Synaptophysin (Syp) is a synaptic vesicle membrane protein, interacting with the SNARE complex, and thus involved in vesicle formation, neurotransmitter release, and synaptic plasticity [62]. Synaptophysin-1 expression level shows no circadian regulation in the cortex, hippocampus, striatum, cerebellum, or thalamus, although it is involved in the resetting of the mammalian circadian clock through the formation of Synaptophysin-Synaptobrevin heterodimers [63, 64]. Chronic sleep restriction (3 h, 5 days a week for 30 days) significantly decreases the level of Syp in the hippocampus but not in the frontal cortex in mice, and long-term sleep deprivation (48 h) reduces the level of Syp in rat hippocampus, but caffeine administration prevents its downregulation [65, 66]. Correlating with these results, Syp showed decreased abundance (−1.42-fold change) after sleep deprivation suggesting downregulated vesicle formation and synaptic transmission. Syntaphilin (Snph) inhibits SNARE complex formation and synaptic vesicle fusion by absorbing free syntaxin-1 and transient overexpression of Snph in cultured hippocampal neurons that significantly reduces neurotransmitter release [67]. The almost 4 times increase of Snph protein abundance (+3.86-fold change) suggests a strongly reduced synaptic transmission after sleep deprivation. Synapsin-3 is primarily localized outside of the synapse but in a lower amount also appears at presynaptic terminals where it forms homo- or heterodimers with other synapsins and acts as a negative regulator of transmission [68]. The heteromers interact with the cytoplasmic surface of the synaptic vesicles and modulate the neurotransmitter release by the regulation of their storage and mobilization within the reserve pool. Its reduced copy number in the course of recovery period (−1.49-fold change) might contribute to the restoration of physiological neurotransmission.

Sleep deprivation impairs synaptic endocytic processes in several ways. In our study, sleep loss led to the downregulation of four interacting proteins of the endocytic vesicles, the clathrin light chain A, clathrin light chain B, amphiphysin, and adaptin ear-binding coat-associated protein 1 (−1.52, −1.37, −1.48, −1.60-fold change, respectively).

Clathrins are the major components of coated vesicles sorting cargo at the cell membrane, trans-Golgi network, and endosomal compartments [69]. At the sites of synapses, the clathrin-mediated endocytosis regulates the internalization of receptors and synaptic transmission [70]. Clathrin is composed of three clathrin heavy chains and three clathrin light chains. The two light chain isoforms, besides their structural roles, contribute to cargo selectivity. Amphiphysin interacts with several proteins associated with clathrin-coated pits during the endocytosis and orchestrates the recruitment and action of effectors in parallel to membrane deformation at sites of membrane fission [71]. The adaptin ear-binding coat-associated protein 1 (NECAP1) is another clathrin accessory protein and a binding partner of amphiphysin and AP-2 (adaptor protein complex 2) and acts as a regulator in the clathrin-mediated recycling of synaptic vesicle proteins. NECAP1 regulates the binding of clathrin to the AP-2 and the coordination of accessory protein recruitment, thus modulating the size and number of endocytic structures. NECAP1 facilitates the conformational change or dephosphorylation of the open form of AP-2 which recycles AP-2 back to its inactive state in the cytosol and renders the complex available for another round of endocytosis [72].

Recovery period affected the abundance of the endocytosis-related synaptic protein syntaxin 12 (+1.56-fold change). Although syntaxins are generally more recognized as components of the SNARE complex, the syntaxin 12 isoform has a pronounced role in the regulation of protein transport between late endosomes and the trans-Golgi network and in controlling the intracellular fate of AMPARs and the endosomal sorting of GRIA2 (glutamate receptor 2) subunit [73, 74]. Its upregulation might be necessary for the restoration of glutamatergic transmission.

### Regulatory Proteins

Secretogranins belong to the functionally conserved granin family of proteins, having roles in the secretory pathway responsible for controlled delivery of peptides, hormones, neurotransmitters, and growth factors [75]. Secretogranin-2 (Scg2) is a preprotein, distributed widely throughout the endocrine and nervous system and enzymatically processed in large dense-core vesicles of neurons to smaller peptides by the proprotein convertase 1 to yield the neuropeptide secretoneurin (SN). SN has roles in the stimulation of neurite outgrowth and neural protection [76]. In our proteomics study, sleep deprivation induced decreased (−1.62-fold change) synaptic protein level of Scg2 in the cerebral cortex suggesting a downregulated delivery of neurotransmitters. The reduced Scg2 abundance is associated with a compromised neurotransmitter secretion, which is in accordance with protein changes observed among the proteins involved in neurotransmitter exocytosis. Calreticulin (Calr) is a lectin-like multifunctional protein, localized in the endoplasmic reticulum. It is involved in regulation of Ca^2+^ homeostasis, influences Ca^2+^-dependent signaling and transcriptional pathways, and is involved in the generation of conformationally competent and functional forms from the newly synthetized, unfolded proteins [77]. Calr plays a role in the modulation of memory formation, consolidation, and retrieval in multiple ways. It is involved in protein synthesis-dependent long-term memory formation, and intracellular calcium dynamics also lead to Ca^2+^-dependent activation of genes such as Calr, which aid calcium homeostasis and neurotransmitter release processes especially in the synaptic area [78]. Function of Calr in sleep has been implicated in *Drosophila melanogaster*, as the mutational effect of *P*-element insertion in Calr gene affects sleep phenotypes [79, 80]. Moreover, Calr binds in a highly specific way to melatonin, an endogenous signal of environmental darkness [81]. During our experiments, Calr showed a decreased (−1.59-fold change) synaptic protein level in the cerebral cortex after sleep deprivation, indicating a compromised memory formation and consolidation, and downregulated neurotransmitter release in the sleep-deprived animals. As a regulator of Ca^2+^ homeostasis, Calr might contribute to sleep regulation in an additional manner. The Ca^2+^-dependent hyperpolarization pathway is believed to be involved in sleep regulation. The sleep-wake cycle affects the activity of kinases downstream of Ca^2+^ signaling and ion channels and kinases acting downstream of calcium signaling regulate the cortical-membrane potential and sleep duration. The sleep-wake state of cortical neurons is partly regulated through the cortical neurons’ intrinsic ability to initiate a slow firing pattern related to the slow-wave oscillation observed in the sleeping brain. The fast and slow Ca^2+^-dependent hyperpolarization pathways are pivotal in generating the SWS firing pattern and regulating sleep homeostasis, respectively [82]. The Ca^2+^-dependent hyperpolarization hypothesis of sleep control proposes that sleep state accompanied with higher hyperpolarization activity can be triggered via Ca^2+^ influx by activating a Ca^2+^-dependent pathway such as calcium/calmodulin-dependent kinase II (CaMKII) a/b to induce NREM sleep [83]. Since Calr binds Ca^2+^ with low affinity but high capacity, its abundance changes might be able to modulate Ca^2+^-dependent hyperpolarization pathways and consequences of Ca^2+^ influx. The fatty acid amide hydrolase (Faah) is a homodimer integral membrane serine hydrolase that belongs to the amidase-signature family and has a complementary cellular localization to the cannabinoid receptor type I (CB_1_). Faah modulates a number of behavioral processes through the hydrolytic inactivation of bioactive fatty acid amides. The hydrolysis terminates the action of several endogenous lipid messengers acting on distinct G protein-coupled receptors, such as the endocannabinoid anandamide (*N*-arachidonylethanolamide) and oleamide [84]. In general, sleep-wake cycle is under the influence of the endocannabinoid system. Blocking of the CB_1_ cannabinoid receptor promotes wakefulness and decreases slow-wave sleep (SWS) and rapid eye movement sleep (REMS), whereas the obstruction of anandamide membrane transporter function enhances sleep [85]. Among the substrates of Faah, anandamide and oleamide are implicated in regulatory mechanisms of sleep. Anandamide enhances sleep through the activation of the CB_1_ receptor. Administration of anandamide leads to a decrease in wakefulness and an enhancement in SWS and REMS. Oleamide affects a wide range of receptors and neurotransmitter systems and, despite not binding to the CB_1_ receptor, has similar pharmacological effect on sleep as anandamide [86]. Knocking-out of *Faah* in mice does not disrupt the homeostatic modulation of sleep, but induces a significant increase in the amount of SWS and intensity of SWS episodes, determined by the high values of SWA, the increase in the duration and decrease in the number of individual episodes of SWS and brief awakenings (BA), and the inverse correlation between SWA and BA. *FAAH* (-/-) mice possess a 15-fold elevated brain level of anandamide and reduced hydrolytic rates for anandamide and oleamide [87]. The reduced expression level of Faah (−1.46-fold change) after the recovery period indicates elevated levels of the Faah substrate anandamide and oleamide, thus a higher activation state of cortical CB_1_ receptors. The predicted higher levels of anandamide and oleamide inhere with a decrease in wakefulness. The supposedly elevated levels of Faah substrates might be responsible factors in longer-term consequences of sleep deprivation exceeding the recovery period. Monoamine oxidase (MAO) is a homodimer flavoprotein enzyme associated with the mitochondrial outer membrane. MAO catalyzes the oxidative deamination of monoamine neurotransmitters and dietary primary and some secondary amines in the brain and peripheral tissues. Among the two isoforms, MAO-A preferentially oxidizes serotonin and noradrenaline, whereas MAO-B preferentially oxidizes phenylethylamine and benzylamine. In most species, dopamine can be oxidized by both isoforms, but in humans, it is exclusively oxidized by MAO-B, while in rodents, only by MAO-A [88]. Levels of the MAO substrate monoamines vary within brain regions and fluctuate with circadian cycle and sleep stages. Monoamines promote wakefulness via a network comprising the thalamus, hypothalamus, basal forebrain, cerebral cortex, and wake-promoting nuclei of the brainstem and inhibit sleep-promoting regions such as the ventrolateral optic area [89]. Generally, monoamine levels are decreased during sleep compared to wakefulness; thus, noradrenaline and serotonin levels decrease from wakefulness to SWS and further decrease during REMS. Sleep deprivation modifies the cycle of monoamine levels in the long run. Monoamine levels increase during sleep deprivation and sometimes, except for serotonin, remain high even during the subsequent recovery sleep [89]. Inhibition of MAO has been used to treat neurodegenerative and psychiatric disorders, and pharmacological intervention with MAO inhibitors has been associated with increased sleep latency, poorer sleep efficiency, and in some cases with insomnia and REMS suppression [90]. The detected downregulation of MAO-B (−1.82-fold change) during the recovery period might account for the long-lasting effect of sleep deprivation on monoamine transmitters and explain their still elevated levels during the recovery period.

### Energetics and Metabolomics

Cytochrome *c* (Cyt *c*) is localized in the cristae of the inner mitochondrial membrane and participates in the mitochondrial electron-transport chain shuttling electrons from Cyt *c* reductase to Cyt *c* oxidase (Cox). Wakefulness is associated with an increased expression and activity of Cox as compared with sleep in multiple brain regions. mRNA for Cox1 subunit is upregulated in rat cortex and *Drosophila* during wakefulness [91–94]. In rats, mRNA level of both the catalytic Cox1 encoded by the mitochondrial genome and the nuclear-encoded Cox4 subunit are increased during wakefulness compared to sleep [95]. It is generally hypothesized that the increased activity of Cox enzyme during wakefulness contributes to the mechanisms providing sufficient amounts of ATP to meet increased neuronal energy demands. In our study, Cycs (Cytochrome c, somatic) showed an increased (+1.36-fold change) protein level after recovery period indicating increased overall Cox enzyme activity ensuring sufficient ATP production at this phase. As generally wakefulness and not sleep is associated with elevated levels of the mitochondrial electron-transport chain proteins, we assume that the prolonged wakefulness during the sleep deprivation period caused extensive increase in Cycs expression and the protein level could not resettle to the baseline level until the end of the recovery period or the elevated expression of the Cycs during the recovery period is needed to serve the energy demand for the widespread molecular reorganization of the synapse. Prkag1, upregulated in our experiment due to sleep deprivation (+1.56-fold change), is the AMP/ATP-binding regulatory subunit of the AMP-activated protein kinase (AMPK). AMPK is energy sensing serine/threonine kinase whose metabolic regulator activity is activated in response to reduction of intracellular ATP levels. The activated AMPK redirects metabolism toward increased catabolism and decreased anabolism through the phosphorylation of key proteins in several pathways, including mTOR complex, lipid homeostasis, glycolysis, and mitochondrial homeostasis and rewires cellular metabolism in a prolonged manner by targeting transcriptional regulators [96]. AMPK plays an important role in linking energy metabolism and the regulation of circadian and sleep homeostasis [97]. ATP levels drastically change during sleep in several brain regions, and sleep deprivation induces a significant reduction in ATP concentration in the frontal cortex and lateral hypothalamus [98]. Owing to the reduced ATP level, 6 h of sleep deprivation increases AMPK activity and the mRNA levels of Ca^2+^/calmodulin (CaM)-dependent protein kinase β (Camk2b), an additional upstream kinase of AMPK in the hypothalamus of mice [99]. Moreover, although AMPK and mTOR regulate different pathways, there are hubs of crosstalk where both converge to regulate cell metabolism. AMPK inhibits mTORC1 through the phosphorylation of Raptor and TSC2. Reciprocally, mTORC1 signaling regulates AMPK as S6K phosphorylates and inhibits AMPK [100]. In consequence of the activated AMPK signaling due to sleep deprivation, all proteins involved in lipid and fatty acid metabolism (Acaca, Decr1, Pccb, Eci2, Acly, and Ephx2) and the majority of the proteins having role in carbohydrate metabolism (Pgm1 and Idh3a) were upregulated in our experiment. In contrast, at the end of the recovery period, all proteins alliable to lipid and fatty acid metabolism (Cpt1a, Faah, Eci1, and Decr1) were downregulated, suggesting that 16 h of recovery period is sufficient to restore the energy balance in the cortex after the prolonged wakefulness. The mitochondrial 2,4-dienoyl-CoA reductase (Decr1) is an auxiliary enzyme of fatty acid beta-oxidation. The enzyme is involved in the metabolism of all unsaturated fatty acids with double bonds originating at even-numbered positions and some unsaturated fatty acids with double bonds originating at odd-numbered positions, regardless of the stereochemical configuration of the double bonds [101]. Interestingly, in our proteomic analysis, we measured an elevated enzyme level (+1.94-fold change) in consequence of sleep deprivation and a decreased protein level (−1.99-fold change) after recovery period. However, so far no specific sleep-related molecular processes have been associated with the enzyme.

## Conclusion

In the present study, we report the effects of sleep deprivation and recovery period on the rat cortical synaptic proteome. The synaptic proteome signature of SD and RP revealed an opposing trend in protein expression changes. The abundance of synaptic proteins has changed to a greater extent in consequence of SD than during RP, and the levels of most of the altered proteins were upregulated during SD, while RP showed the opposite tendency. Enrichment analysis of biological processes identified proteins involved in key synaptic processes, like signal transduction, synaptic assembly, or synaptic transmission and highlighted a prominent role of GABAergic signaling in sleep regulation. An enrichment was apparent in favor of synaptic transmission- and excitability-related proteins among the sleep-related ones, while the wake-related proteins are associated with synaptic excitability and inhibition in a nearly equal ratio. Studying the molecular consequences of SD and RP by analyzing the 117 significantly altered proteins in the two experimental settings we found evidence for both synaptic homeostasis and synaptic embossing theories. We report on the involvement of highly abundant scaffold proteins in the structural rearrangement and on the changes of several well-known proteins playing role in the release or reuptake of neurotransmitters. Moreover, our data further highlight the importance of regulatory and metabolomic pathways, which control aspects of wake or sleep state or supply energy for the synaptic molecular transformations.

## Supplementary Information

Below is the link to the electronic supplementary material.

Supplementary file1 (XLSX 21 KB)

Supplementary file2 (XLSX 16 KB)

## Data Availability

Electronic supplementary material is available for the manuscript.

## Abbreviations

SD: Sleep deprivation
RP: Recovery period
SHY: Synaptic homeostasis hypothesis
LC-MS/MS: Liquid chromatography-tandem mass spectrometry
DTT: Dithiothreitol
PTM: Posttranslational modification
ACSF: Artificial cerebrospinal fluid
ER: Endoplasmic reticulum
CNS: Central nervous system
LTP: Long-term potentiation
LTD: Long-term depression
GHB: γ-Hydroxybutyric acid
SWS: Slow-wave sleep
REMS: Rapid eye movement sleep
BA: Brief awakenings
MAGUK: Membrane-associated guanylate cyclase kinase
NMDAR: N-methyl-D-aspartate receptor
AMPAR: α-Amino-3-hydroxy-5-methyl-4-isoxazoleproprionic acid receptor
FA: Formic acid
can: Acetonitrile
